# Synergistic effect of quinary molten salts and ruthenium catalyst for high-power-density lithium-carbon dioxide cell

**DOI:** 10.1038/s41467-019-14121-1

**Published:** 2020-01-23

**Authors:** Kyungeun Baek, Woo Cheol Jeon, Seongho Woo, Jin Chul Kim, Jun Gyeong Lee, Kwangjin An, Sang Kyu Kwak, Seok Ju Kang

**Affiliations:** Department of Energy Engineering, School of Energy and Chemical Engineering, Ulsan National Institute of Science and Technology (UNIST), Ulsan, 44919 Republic of Korea

**Keywords:** Carbon capture and storage, Batteries, Materials for energy and catalysis

## Abstract

With a recent increase in interest in metal-gas batteries, the lithium-carbon dioxide cell has attracted considerable attention because of its extraordinary carbon dioxide-capture ability during the discharge process and its potential application as a power source for Mars exploration. However, owing to the stable lithium carbonate discharge product, the cell enables operation only at low current densities, which significantly limits the application of lithium-carbon dioxide batteries and effective carbon dioxide-capture cells. Here, we investigate a high-performance lithium-carbon dioxide cell using a quinary molten salt electrolyte and ruthenium nanoparticles on the carbon cathode. The nitrate-based molten salt electrolyte allows us to observe the enhanced carbon dioxide-capture rate and the reduced discharge-charge over-potential gap with that of conventional lithium-carbon dioxide cells. Furthermore, owing to the ruthernium catalyst, the cell sustains its performance over more than 300 cycles at a current density of 10.0 A g^−1^ and exhibits a peak power density of 33.4 mW cm^−2^.

## Abstract (web summary)

Lithium-carbon dioxide cells are challenging due to the sluggish electron transfer in the Lithium carbonate in aprotic electrolyte. Here, the authors report synergistic effect of molten salt electrolyte and Ruthenium catalyst to enhance the electrochemical performance of Lithium-carbon dioxide batteries

## Introduction

Rechargeable alkali metal–gas cells have attracted considerable interest for high-energy-density storage systems because of their ultralightweight air cathodes such as O_2_ and CO_2_ gases^1–8^. When directly using the lightest Li metal anode, a specific energy of 3860 mAh g^−1^ can be achieved with an O_2_ cathode, which undoubtedly solves the energy constraints of the current Li-ion batteries^1–3^. However, an unwanted side reaction elicited by the aprotic electrolyte and the carbon current collector significantly erodes the Li–O_2_ battery performance^9–11^. This limitation has ultimately led to intensive research on electrochemically inert electrolytes and novel cathode materials. Among the many innovative proposed material candidates, the nitrate-based molten salt electrolyte and the porous Au cathode exhibited a promising oxygen-evolution reaction (OER)/oxygen-reduction reaction (ORR) ratio, measured by differential electrochemical mass spectrometry (DEMS)^12,13^. The key finding behind these studies is that the stable components against the Li–O_2_ electrochemical reaction effectively minimize the detrimental side reactions and allow operation of the battery cycle without significant degradation.

However, there is still one cumbersome problem: the accumulation of the parasitic product Li_2_CO_3_ during the discharge–charge processes^9,14^. In particular, it is well known that the stable nature of Li_2_CO_3_ inevitably induces dead space in the cathode side, which eventually causes decreased cycle ability and increased charge potential. In order to cope with this fatal issue, paradoxically, researchers have successfully proposed a Li–CO_2_ cell and suggested the importance of its application as both a rechargeable secondary battery and CO_2_ capture device to retard global warming^5,15–22^. The proposed CO_2_ reduction reaction (CO_2_RR) in the Li–CO_2_ battery is based on the following electrochemical reaction: 4Li^+^ + 3CO_2_ + 4e^−^ → 2Li_2_CO_3_ + C (2.80 V vs. Li/Li^+^)^5^. During the galvanostatic discharge process, the Li–CO_2_ cell needs 4/3 electron to capture a single CO_2_ gas molecule and produces the 2Li_2_CO_3_ and amorphous carbon on the cathode side, indicating that the electron-to-CO_2_ ratio (e^–^/CO_2_) is 1.33.

Although the Li–CO_2_ cell effectively captures CO_2_ gas during the discharge process, the high charge overpotential caused by the insulating and insoluble characteristics of Li_2_CO_3_ in the aprotic electrolyte should be reduced to prevent the severe parasitic reaction^7,15,16,23^. Therefore, most recent research on Li–CO_2_ cells has focused on developing air-breathing cathodes such as metal catalysts, mediators, and metal oxide materials for reducing the charge overpotential and increasing the cycle ability^8,18,24–28^. However, because of the sluggish electron transfer in the Li_2_CO_3_ insulator, most of the reported studies investigated Li–CO_2_ cells with mild current densities (Supplementary Table 1), which are not appropriate for high-performance battery applications, the limiting factor for the CO_2_ capture rate; thus, enhancement of the rate performance certainly is advantageous to facilitate practical future battery and CO_2_ storage applications of Li–CO_2_ cell.

Here, we report the demonstration of a high-power-density Li–CO_2_ cell based on a quinary-molten salt electrolyte containing Ru nanoparticles on the carbon cathode. The employed nitrate-based quinary-molten salt allows high-temperature operation of the Li–CO_2_ battery, which reduces the discharge–charge overpotential. Moreover, the presence of Ru nanoparticles on the carbon cathode prepared by the Joule heating method further improves the electrochemical characteristics of the Li–CO_2_ cell, resulting in a long cycle life of more than 300 cycles at a high-current density of 10.0 A g^−1^. In addition, a high-power-density of 33.4 mW cm^−2^ is successfully achieved; this is a potentially decisive factor for next-generation high-rate rechargeable Li–CO_2_ batteries and efficient CO_2_-capture electrochemical cells.

## Results

### Aprotic electrolyte-based Li–CO_2_ cell

**Fig. 1:**
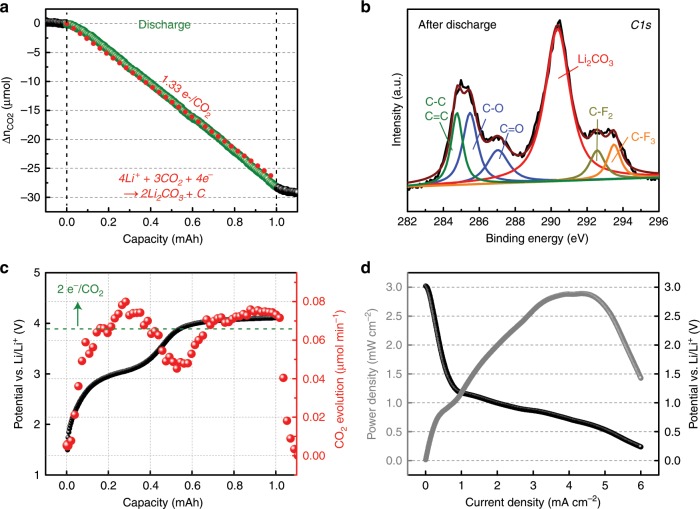
Electrochemical characterizations of Li–CO_2_ cell by using an aprotic electrolyte. **a** Plot of CO_2_ gas consumption during the galvanostatic discharge (200 μA). The Li–CO_2_ cell contains 1 M LiTFSI in TEGDME electrolyte. The red dots indicate the ideal electron-to-CO_2_ ratio of 1.33. **b** C1s XPS result of the carbon cathode after 1-mAh discharge. **c** Galvanostatic charge plot and the corresponding DEMS results of the Li–CO_2_ cell containing 1 M LiTFSI in TEGDME electrolyte. The green dots correspond to the theoretical amount of CO_2_ evolution. **d** Polarization and power-density curves of 1 M LiTFSI in TEGDME with a scan rate of 0.01 mA s^−1^.

Following recent Li–CO_2_ battery studies (Supplementary Table 1), we first fabricated a cell consisting of a Li metal anode, carbon cathode, and 1 M lithium bis(trifluoromethanesulfonyl)imide (LiTFSI) in tetraethylene glycol dimethyl ether (TEGDME) electrolyte to monitor the CO_2_RR during the galvanostatic discharge process by using pressure decay measurement. As shown in Fig. 1a, the linear drop of consumed CO_2_ molecules indicates that the value of the electrons per CO_2_ ratio is ~1.32, which agrees well with the previously proposed discharge electrochemical reaction of 4Li^+^ + 3CO_2_ + 4e^−^ → 2Li_2_CO_3_ + C (2.80 V vs. Li/Li^+^)^5^. In addition, the C1s X-ray photoelectron spectroscopy (XPS) spectra of a 1 mAh discharged carbon cathode presented a strong peak at 290 eV as shown in Fig. 1b, revealing the formation of Li_2_CO_3_ during the discharge process (Supplementary Fig. 1)^8^. However, the amount of CO_2_ evolution during the charge process measured by DEMS in Fig. 1c shows that the electrochemical reaction clearly does not follow the discharge reaction of 4Li^+^ + 3CO_2_ + 4e^−^ → 2Li_2_CO_3_ + C (2.80 V vs. Li/Li^+^)^5^. In particular, the different electrochemical reactions and multiple charging plateaus during the charging process have been reported^5,7^. For instance, Qiao et al. showed that there are several different pathways to decompose the stable Li_2_CO_3_^5^. Depending on the applied current and charging voltage, the electrons per CO_2_ can be either 1.5 or 2.0 e^−^/CO_2_ during the charging process, implying an irreversible electrochemical reaction in the Li–CO_2_ battery. In addition, Zhang et al. observed multiple plateaus with fluctuating evolution rates of CO_2_ gas during the charging process^7^. Nevertheless, to further understand this irreversible electrochemical reaction, we employed a carbon isotope (^13^C) cathode and performed linear-sweep voltammetry with the corresponding DEMS measurements (Supplementary Fig. 2). Although both ^12^CO_2_ and ^13^CO_2_ (mass weight of 45) are evolved at the same potential, the trace of the mass weight of 45 may not originate from ^13^CO_2_ because a previous study clearly showed that the decomposition of TEGDME generates fragment-45 owing to the generation of superoxide radicals during the decomposition of Li_2_CO_3_; these results are in good agreement with ours^29^. In addition, low peak power density of 2.9 mW cm^−2^ (Fig. 1d) and the evolution of H_2_ and CO_2_ gases at the potentials of 4.2 and 4.7 V in a fresh cell before discharging (Supplementary Fig. 3) clearly reveal further disadvantages of using aprotic solvent, which ultimately leads us to attempt an alternative electrolyte for high-performance Li–CO_2_ cell. It is noted that the H_2_ evolution from the fresh cell is presumably due to the parasitic reaction between the aprotic electrolyte and the Li metal^30,31^.

### Molten salt-based Li–CO_2_ cell

**Fig. 2:**
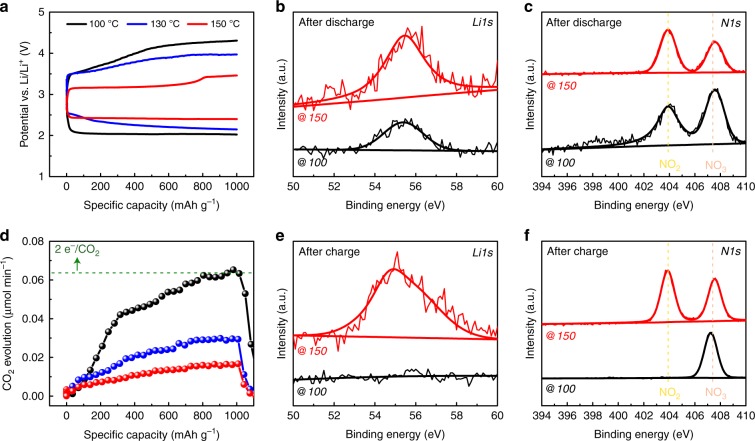
Characterizations of Li–CO_2_ battery by using quinary-molten salt electrolyte. **a** Galvanostatic discharge and charge profiles of a Li–CO_2_ battery with quinary-molten salt electrolyte at different operating temperatures (100–150 °C) at current density of 0.4 A g^−1^. **b**–**c** High-resolution XPS Li1s (**b**) and N1s (**c**) spectra of the carbon cathode after 1000 mAh g^−1^ discharge. **d** DEMS result of the Li–CO_2_ cell containing quinary-molten salt electrolyte at different operating temperatures during charge process in **a**. **e**–**f** The green dots correspond to the theoretical amount of CO_2_ evolution. High-resolution XPS Li1s (**e**) and N1s (**f**) spectra of the carbon cathodes after 1000 mAh g^−1^ discharge and charge processes. The black and red lines indicate the results at operating temperatures of 100 and 150 °C, respectively.

In order to reduce the overpotential at high-current densities during the charge process, we prepared a cell with nitrate-based quinary-molten salt for an aprotic solvent-free electrolyte. The low eutectic melting temperature of the quinary-molten electrolyte allows us not only to perform a systematic study of the Li_2_CO_3_ decomposition process in a wide temperature range from 100 to 150 °C but also to potentially use for CO_2_ capture from the high-temperature power plant flue gas (Supplementary Fig. 4)^32^. As shown in the galvanostatic discharge–charge profiles in Fig. 2a, we observed that the operating temperature plays a critical role in reducing the discharge and charge overpotential of the Li–CO_2_ battery. For instance, the Li–CO_2_ cell at 150 °C exhibits a discharge–charge potential gap of 0.7 V, whereas the cell at 100 °C exhibits an ~2.1 V potential gap. In addition, the multiple charging plateaus may be due to the parasitic reaction between the discharge product and the carbon surface, which we will discuss later in Fig. 4. The pressure-drop measurement and Li1s XPS analysis further verify the electrochemical reaction of Li–CO_2_ cell in the nitrate-based electrolyte (Fig. 2b and Supplementary Fig. 5). Although we observed a strong signal at 55.3 eV for the Li_2_CO_3_ compound, the measured electron-to-CO_2_ ratio of 2.0 is in contrast to the aprotic electrolyte-based Li–CO_2_ cell in Fig. 1. This different electrochemical reaction can be explained by the previous nitrate molten electrolyte-based Li–O_2_ battery studies because the regeneration of NO_3_^−^ from NO_2_^−^ leads to alter the conventional electrochemical reaction of Li–O_2_ cell^33–35^. In the case of nitrate molten salt in Li–CO_2_ cell, N1s XPS analysis in Fig. 2c shows the existence of NO_2_ compound after the discharge process, which is evidence that the nitrate anion is involved in the electrochemical reaction and altered the electrochemical reaction and discharge potential of Li–CO_2_ cell (Supplementary Table 2, Supplementary Figs. 6–7)^33–35^. However, during the charging process, the DEMS results in Fig. 2d found that the CO_2_ evolution rates varied with operating temperature. The CO_2_ evolution rate increases with decreasing operating temperature, ranging from ~2.0 e^−^/CO_2_ at 100 °C to ~6.0 e^−^/CO_2_ at 150 °C. The value of 0.02 μmol min^−1^ at 150 °C during the galvanostatic charging process indicates that the electrochemical reaction is irreversible and different from the discharge process. In addition, the shifted Li_2_CO_3_ peak and the remaining NO_2_ compound in the XPS analysis after discharge and charge cycles at 150 °C suggests the formation of a new adduct during the charging process that significantly reduces CO_2_ evolution (Fig. 2e–f). It is noted that the molten salt electrolyte exhibits monotonically increasing CO_2_ rate that may be due to the enhanced CO_2_ solubility at high temperature. We observed a sharp increase in the gas evolution after CO_2_ gas saturation by using a deep discharged cell that also provides electron-to-CO_2_ ratio in the molten electrolyte (Supplementary Fig. 8). Furthermore, we performed linear-sweep voltammetry and carried out the galvanostatic charging process with the corresponding DEMS measurements at 100 °C to confirm the contribution of CO_2_ evolution by using the ^13^CO_2_–^12^C and ^12^CO_2_–^13^C systems (Supplementary Fig. 9). Although we observed evidence of carbon decomposition in the linear-sweep voltammetry results for both systems, the galvanostatic charging process with the corresponding DEMS measurements showed marginal CO_2_ evolution from the carbon cathode, which indicates that CO_2_ evolution predominantly occurs from Li_2_CO_3_ decomposition at 100 °C (Supplementary Fig. 10).

### Reaction mechanism of Li_2_CO_3_ decomposition

**Fig. 3:**
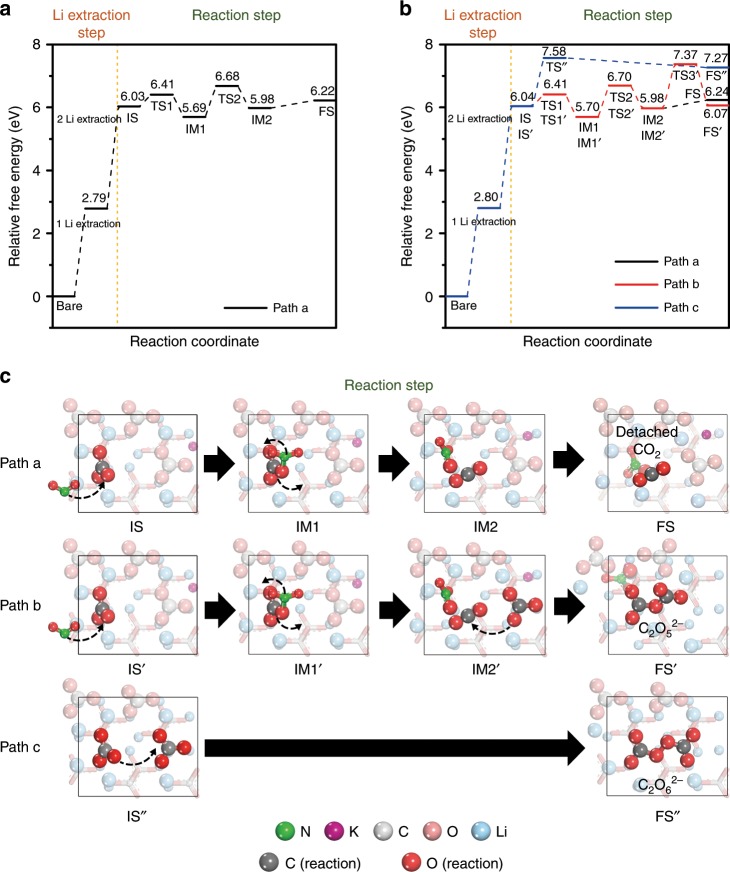
Reaction mechanism of Li_2_CO_3_ decomposition. **a** Reaction coordinate of one possible path a to produce CO_2_ and NO_3_^−^ (black line) at 100 °C. **b** Reaction coordinate of three plausible pathways (i.e., path a, path b to produce C_2_O_5_^2−^ and NO_3_^−^ (red line), and path c to produce C_2_O_6_^2−^ (blue line)) at 150 °C. **c** Optimized configurations on three plausible pathways for the reaction step corresponding to (**a**) and (**b**). IS, IM, and FS in each reaction mechanism represent the initial state, intermediate state, and final state, respectively. The yellow dotted line is the boundary between the Li extraction step and the reaction step, and the numbers represent the relative free energies based on those of bare surface in (**a**) and (**b**). Nitrogen, potassium, carbon, oxygen, and lithium atoms are colored in green, purple, light gray, pink, and sky blue. For a clear view, the carbon, oxygen, and lithium atoms, which participate in the reaction, are colored in dark gray, red, and blue. Arrow dotted lines represent the movement of molecules from state to state. For a clear view, the molecules, except reacting molecules, were made to be translucent in (**c**).

Although the exact electrochemical reaction remains unclear, we examined the Li_2_CO_3_ decomposition mechanism by the density functional theory (DFT) calculation to explain the variation of generating the amount of CO_2_ depending on the operating temperature (i.e., 100 °C and 150 °C) of Li–CO_2_ cells (see the “Supplementary Methods” section and Supplementary Fig. 11). The Li_2_CO_3_ decomposition mechanism under implicit quinary-molten salt condition was divided into the electrochemical reaction step, where Li ion is extracted by the charge potential, and the thermodynamic reaction step, where the carbonate on the surface participates in the reaction. We compared the Li extraction energy and activation energy of CO_2_ formation reaction by NO_2_^−^ to determine the reaction priority (Supplementary Fig. 12). Since the Li extraction energy (i.e., 2.79 and 3.24 eV for the first and second Li extraction, respectively) was lower than the activation energy of CO_2_ formation reaction (i.e., 4.01 eV), it was predicted that the CO_2_ formation reaction could occur after the Li extraction reaction. Thus, we suggest path a (Li_2_CO_3_ + NO_2_^−^ → 2Li^+^ + CO_2_ + NO_3_^− ^+ 2e^−^) for the decomposition mechanism of Li_2_CO_3_ at 100 °C (Fig. 3a–c). In path a, after the two Li atoms were extracted, carbonate ion reacted with NO_2_^−^ to produce [CO_3_NO_2_]^−^ at the first intermediate state (IM1). From IM1 to IM2, a bridge O atom bonded to C and N atoms was moved to form NO_3_^−^ and produce CO_2_. Then, CO_2_ was desorbed from the surface in the final state (FS). The full-charge N1s XPS analysis showed no peak of NO_2_^−^ because of the generation of NO_3_^−^ as we conjectured (Fig. 2f). At 150 °C, as shown in Fig. 3b–c, the Li_2_CO_3_ decomposition mechanism initially followed the same reaction process of path a. However, after CO_2_ and NO_3_^−^ are formed on the surface (IM2’) in path b (2Li_2_CO_3_ + NO_2_^−^ → 4Li^+^ + C_2_O_5_^2−^ + NO_3_^− ^+ 2e^−^), CO_2_ could react further with the adjacent carbonate to form C_2_O_5_^2−^ (FS’). Separately, the unstable carbonate could react with the adjacent carbonate to form C_2_O_6_^2−^ (FS”) in path c (2Li_2_CO_3_ → 4Li^+^ + C_2_O_6_^2−^ + 2e^−^), where NO_2_^−^ was not used as the reactant in the Li_2_CO_3_ decomposition mechanism. The three paths in the reaction mechanisms predicted to be occurred at 150 °C were consistent with experimental results, where NO_2_^−^ and NO_3_^−^ presented on the surface and a small amount of CO_2_ was released (Fig. 2d–f). We speculated that the thermal energy at the higher temperature could promote the reactions of paths b and c; the activation energies of the two mechanisms (i.e., 1.39 eV for path b and 1.54 eV for path c at 150 °C) were higher than the activation energy for the mechanism to produce CO_2_ gas (i.e., 0.99 eV for path a at 100 °C). Interestingly, CO_2_ was favored in the adsorbed state considering the endothermic heat of reaction from IM2 to FS in path a without a transition state. This also could be a reason for CO_2_ to undergo the reaction step from IM2 to FS at 150 °C. All of the optimized configurations in each reaction mechanism are depicted in Supplementary Fig. 13. It should be noted that because the proposed pre-equilibrium electrochemical reactions are not the complete reaction mechanism of the charge process, the generation of the short-lived intermediate C_2_O_6_^2−^ produces new adducts, resulting in irreversible CO_2_ evolution at 150 °C in the DEMS measurements in Fig. 2.

### Electrochemical performance of Li–CO_2_ cell

**Fig. 4:**
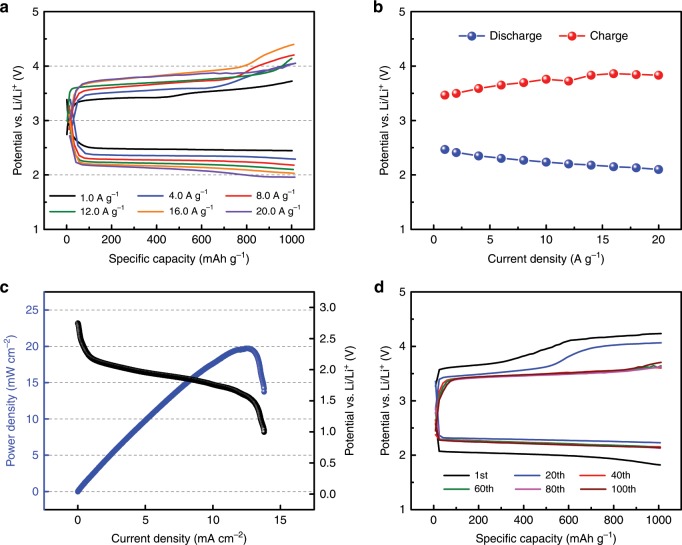
Electrochemical performance of Li–CO_2_ cell with quinary-molten salt electrolyte. **a** Galvanostatic discharge–charge profiles of the Li–CO_2_ battery with quinary-molten salt electrolyte at different current densities from 1.0 to 20.0 A g^−1^ at 150 °C. **b** Plots of discharge–charge overpotential measured at 500 mAh g^−1^ as a function of current density. **c** Plots of operating voltage and power density versus current density of the Li–CO_2_ battery at 150 °C with scan rate of 0.01 mA s^−1^. **d** Galvanostatic discharge–charge profiles of a Li–CO_2_ cell with 2.0 A g^−1^ current density over up to 100 cycles.

To investigate the high-current performance of the quinary-molten salt electrolyte, we evaluated the galvanostatic discharge–charge characteristics of the Li–CO_2_ cell under a current-density range of 1.0–20.0 A g^−1^ (Fig. 4a). As shown in the plots, the discharge–charge overpotentials were dominantly affected by the applied current densities. For instance, the Li–CO_2_ battery at an applied current density of 1.0 A g^−1^ had the lowest discharge–charge potential gap of 1.0 V, whereas that at an applied current of 20.0 A g^−1^ showed the highest potential gap of 1.7 V (Fig. 4b). Although the discharge–charge overpotential gap monotonically increases with increasing applied current density, the stable discharge–charge profiles at 20.0 A g^−1^ clearly suggest that the high-temperature operation of quinary-molten salt at 150 °C efficiently enhances the rate performance of the Li–CO_2_ battery, which is one of the desired battery characteristics and also has the advantage of capturing CO_2_ gas from power plants because the high-current density increases the CO_2_ capture rate (Supplementary Fig. 14). In addition, we observed a high peak power density of 19.7 mW cm^−2^ from the quinary-molten salt-based Li–CO_2_ cell (Fig. 4c); this value is approximately seven times higher than that of the conventional Li–CO_2_ battery with 1 M LiTFSI in the TEGDME electrolyte (Fig. 1d). We also measured the power density with another ternary-molten salt (37 mol% LiNO_3_, 39 mol% KNO_2_, and 24 mol% CsNO_3_) electrolyte at 150 °C (Supplementary Fig. 15). Although the peak power density of 16.2 mW cm^−2^ is slightly lower than that of the quinary-molten salt electrolyte, the ternary-molten salt also increases the electrochemical performance in the Li–CO_2_ battery. The Li–CO_2_ battery with quinary-molten salt further allows us to observe the long-term cycle capability. As shown in Fig. 4d, the results show stable discharge–charge plots over 100 cycles at a high-current density of 2.0 A g^−1^. We observed that the charge overpotential decreases as the number of cycles increases in Fig. 4d. One plausible explanation is the parasitic reaction between Li_2_CO_3_ and the carbon defect sites. Because most metal–gas batteries use the capacity cutoff for cycle measurement, a discharge product is formed on a fresh carbon surface every cycle, which causes a parasitic reaction and multiple plateaus during the charging process. Thus, to mitigate the effect of the carbon surface, we also monitored the second cycle after the potential cutoff operation at the first cycle and observed a single charging plateau, indicating that the carbon surface is important for reducing the parasitic reaction during the charging process (Supplementary Fig. 16).

### Synthesis of Ru nanoparticles by using Joule heating

**Fig. 5:**
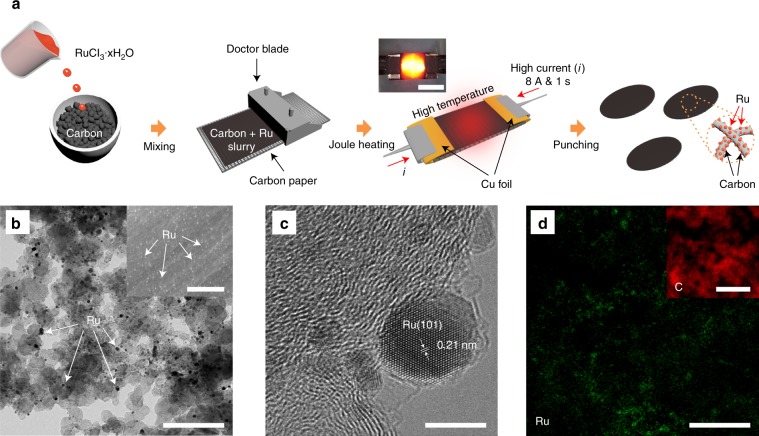
Joule heating induced Ru nanoparticles. **a** Schematic illustration of the fabrication procedure of Ru nanoparticles on the carbon cathode by using the Joule heating method. The inset shows a photograph of light emitting from the carbon cathode during Joule heating. Scale bar is 1 cm. **b** Bright-field TEM micrograph and SEM image (inset) of the carbon cathode with Ru nanoparticles after applying 8 A for 1 s. Scale bars are 200 nm. **c** High-resolution TEM image of Ru nanoparticles on the carbon cathode. Scale bar is 5 nm. **d** EDS mapping of Ru and C (inset) elements of the TEM image in (**b**). Scale bars are 200 nm.

Further enhancement of the Li–CO_2_ cell can be achieved by a carbon cathode with Ru catalyst, because previous reports of Li–CO_2_ batteries by using aprotic electrolytes indicate that the cathode catalyst efficiently promotes the decomposition of Li_2_CO_3_^18,24^. As schematically shown in Fig. 5a, RuCl_3_ in H_2_O solution was mixed thoroughly with Super P carbon powder by a Thinky mixer for 10 min; then, the composite slurry was coated onto the carbon paper to apply the high current. In particular, the Joule heating method allows us not only to homogeneously disperse the Ru nanoparticles with a controlled size but also to reduce the thermal decomposition time of RuCl_3_^36–38^. After a systematic study of Ru nanoparticles with various particle sizes and populations (Fig. 5b (inset) and Supplementary Fig. 17), we found well-developed Ru nanoparticles on the carbon cathode from the optimum conditions of 2:1 weight ratio (Super P:Ru) slurry at 8 A for 1 s. A high-resolution transmission electron microscopy (HR-TEM) measurement further showed well-dispersed Ru particles on the carbon cathode (Fig. 5b). The magnified image in Fig. 5c shows the fringes of the crystalline structure of Ru nanoparticles, where the *d*-spacing of 0.21 nm represents the (101) plane of the Ru crystal^39^. Moreover, the energy-dispersive spectroscopy (EDS) mapping of Ru further supports the well-dispersed Ru nanoparticles on the carbon cathode surface in Fig. 5d (inset image is the mapping of the carbon element), confirming that the Joule heating method is a simple but potent way to develop the Ru catalyst, making it a suitable cathode for Li–CO_2_ batteries without a polymeric binder^40^.

### Synergistic effect of the molten salts and Ru nanoparticles

**Fig. 6:**
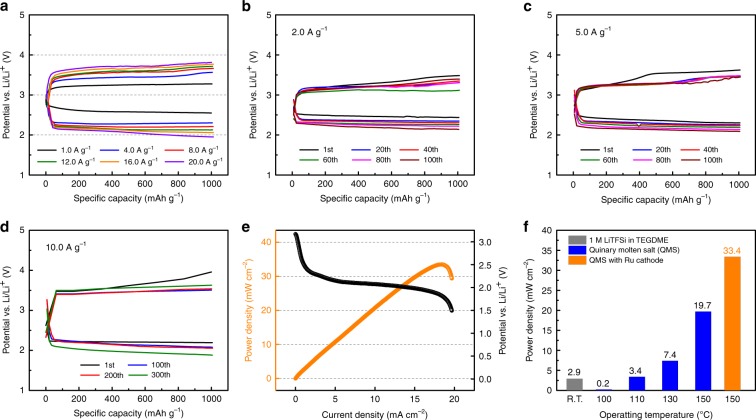
Electrochemical performance of Li–CO_2_ cell by using molten salt and Ru nanoparticle. **a** Galvanostatic discharge–charge profiles of the Li–CO_2_ battery with quinary-molten salt electrolyte with a Ru nanoparticle cathode at different current densities from 1.0 to 20.0 A g^−1^ at 150 °C. Cycling performance of the Li–CO_2_ battery at current rates of 2.0 A g^−1^ (**b**), 5.0 A g^−1^ (**c**), and 10.0 A g^−1^ (**d**). **e** Polarization and power-density curves of quinary-molten salt electrolyte with the Ru nanoparticle cathode with a scan rate of 0.01 mA s^−1^. **f** Plots of peak power density of 1 M LiTFSI in TEGDME (gray); quinary-molten salt at 100, 110, 130, and 150 °C (blue); quinary-molten salt electrolyte with a Ru nanoparticle cathode at 150 °C (orange).

To evaluate the synergistic effect of the quinary-molten salts and Ru nanoparticles on the carbon cathode on the Li–CO_2_ cell performance, we performed galvanostatic discharge–charge measurements with current density ranging from 1.0 to 20.0 A g^−1^ at 150 °C (Fig. 6a). The Ru nanoparticles on the carbon cathode were observed to further reduce the overpotential with a high discharge capacity to form a Li_2_CO_3_ discharge product (44,000 mAh g^−1^ at 10.0 A g^−1^ (Supplementary Figs. 18–19)). Although the proposed discharge reaction shows continuous consumption of NO_3_^−^ during the discharge process, highly concentrated NO_3_^−^ in the molten salt electrolyte does not significantly alter the electrochemical performance during the discharge process. In addition, the cell operates even at a high-current density of 20.0 A g^−1^ and enhances the CO_2_-capturing capacity, in contrast to the aprotic electrolyte-based Li–CO_2_ battery with a Ru catalyst (Supplementary Fig. 20)^24^. We observed a sufficiently stable cycle capability of the Li–CO_2_ battery at a current density at 2.0 A g^−1^ (Fig. 6b), 5.0 A g^−1^ (Fig. 6c), and 10.0 A g^−1^ (Fig. 6d) at 150 °C. In particular, the cells are sustained for over 300 cycles at 10.0 A g^−1^ without significant alteration of the voltage potential. Moreover, the Ru nanoparticles in quinary-molten salt exhibited a peak power density approximately two times that of quinary-molten salt without Ru nanoparticles, indicating that the synergistic effect of Ru nanoparticles further reduces the energy barrier during the electrochemical reaction of the Li–CO_2_ battery (Fig. 6e). In particular, we theoretically revealed that the addition of the Ru surface induced the electron transfer from CO_2_^−^ to Ru particles to stabilize CO_2_^−^, reducing the energy of the thermodynamic barrier (i.e., overpotential) (Supplementary Figs. 21–23). Consequently, the power density of Li−CO_2_ battery was enhanced. As summarized in Fig. 6f, the peak power density of 33.4 mW cm^−2^ is 13 times higher than that of the conventional Li–CO_2_ battery with the aprotic electrolyte (Supplementary Figs. 24–25), suggesting that the quinary-molten salts and the Ru nanoparticles on the carbon cathode make the Li–CO_2_ cell a feasible high-performance CO_2_ capture and energy-storage system.

## Discussion

We have demonstrated a high-performance Li–CO_2_ cell based on the quinary-molten salt electrolyte with Ru nanoparticles on the carbon cathode. From the systematic DEMS investigation with in-depth theoretical investigation, we suggested a newly proposed decomposing reaction mechanism of the Li_2_CO_3_ compound in the nitrate-based molten salt at a high temperature. In addition, the synergistic effect of the quinary-molten salt and the well-distributed Ru catalyst on the carbon cathode allowed us to observe high-rate performance and long-term cycle capability over more than 300 cycles without significant alteration. The Li–CO_2_ battery ultimately achieved the highest peak power density of 33.4 mW cm^−2^, confirming that the Li–CO_2_ cell is suitable as a high-current-rate rechargeable battery and a high-rate CO_2_ capture device.

## Methods

### Preparation of nitrate-based molten salt electrolyte

Lithium nitrate (LiNO_3_), potassium nitrate (KNO_3_), potassium nitrite (KNO_2_), sodium nitrate (NaNO_3_), calcium nitrate (Ca(NO_3_)_2_), and cesium nitrate (CsNO_3_) salts were purchased from Sigma-Aldrich (USA), with purity $\ge$99%, and vacuum-dried at 60 °C for 24 h. We used a specific eutectic composition of 15 mol% LiNO_3_, 30 mol% KNO_3_, 30 mol% CsNO_3_, 10 mol% NaNO_3_, and 15 mol% Ca(NO_3_)_2_ for the quinary-molten salt, and 37 mol% LiNO_3_, 39 mol% KNO_2_, and 24 mol% CsNO_3_ for the ternary-molten salt. The mixture was heated in a ceramic crucible with a torch^32^. The glass microfiber (GF) separator (GF/C, Whatman, UK) with diameter 16 mm was then dipped in the molten eutectic salt (eutectic temperatures, *T*_e_: 75 and 98 °C for the quinary and ternary-molten salts, respectively, Supplementary Fig. 4) to infuse the GF separator and cooled to room temperature. The infused GF separator was dried at ~50 °C in vacuum in an oven for 12 h and transferred into an Ar-filled glove box. The mass of the infused electrolyte was ~160 mg.

### Synthesis of Ru nanoparticles

Ruthenium(III) chloride hydrate (RuCl_3_·xH_2_O, Sigma-Aldrich (USA)) solution (50 mg ml^−1^ in H_2_O) was mixed with carbon (Super P, Timcal (Imerys Graphite & Carbon), Switzerland) at a weight ratio of 2:1 (Super P:Ru) by a Thinky mixer for 10 min. The homogenously mixed carbon with Ru solution was coated onto the carbon paper (AvCarb P50, FuelCellStore (USA)) using a doctor blade. The coated electrode was then dried at 120 °C in a vacuum oven for 6 h to completely eliminate the residual solvent. The RuCl_3_–carbon electrodes were treated by electric Joule heating to form Ru nanoparticles on the carbon. To perform the Joule heating, the sample was connected to copper electrodes and electrically connected to an external power source (Regulated DC Power Supply TDP-3010B, TOYOTECH, Korea) in an argon-filled glove box. A current pulse of 8 A was applied through the sample, which created a Joule heating time of 1 s. The loading mass of Ru on Super P (Ru + Super P) was ~0.45 mg cm^−2^.

### Li–CO_2_ cell assembly

For the preparation of the cathode for the aprotic electrolyte, the air cathode was fabricated using a mixture of Ketjen black carbon (EC-300J) or a carbon isotope (Carbon-^13^C, Sigma-Aldrich (USA)) with a 60 wt% polytetrafluoroethylene (PTFE, Sigma-Aldrich, USA) binder at a weight ratio 9:1 (carbon:binder). The mixture was dispersed in water solution and cast on a SUS mesh (stainless-steel mesh, Shinmyung Science Inc.) current collector. The mixture was dried overnight at 120 °C in a vacuum oven to eliminate the residual solvents. The cathode-loading mass was ~0.3 mg cm^−2^.

For the preparation of cathodes for the quinary-molten salt electrolyte, Super P porous carbon was homogeneously mixed with a 60 wt% PTFE binder (weight ratio = 9:1) in a water solution. A P50 carbon cathode was then coated with the Super P mixture using a doctor blade, and the coated electrode was then dried at 120 °C in a vacuum oven for 6 h to completely eliminate the residual solvent. The loading mass weight of Super P carbon was ~0.45 mg cm^−2^.

In all, 1 M LiTFSI in the TEGDME was purchased from Enchem (Korea) and stored in an Ar-filled glove box with moisture and oxygen levels of <1 ppm. The Li–CO_2_ cell was assembled into a 2032-format coin cell (Hohsen, CR2032, Japan) containing 30 holes with diameters of 1 mm for the Li–CO_2_ electrochemical test. The pure-Li metal anode with thickness of 300 μm was purchased from FMC (Korea) and used as received. The GF separator was dried overnight at 150 °C in a vacuum oven. The cell fabrication was carried out in an Ar atmosphere (H_2_O and O_2_ < 1 ppm). A quinary- or ternary-molten-salt-based Li–CO_2_ cell was fabricated by placing an electrolyte-infused GF separator on Li metal having a diameter of 11 mm using a 2032 coin cell with 30 holes with diameters of 1 mm. Li metal/electrolyte-infused GF separator/carbon cathode cells were pressurized by using a crimping machine.

### Material characterization

The morphology of the Ru nanoparticles on the carbon cathode was characterized using high-resolution transmission electron microscopy (HR-TEM; JEM-2100F, JEOL, Japan) at an accelerating voltage of 200 kV. The discharge products for carbon with and without the Ru nanoparticle cathode were analyzed by using a scanning electron microscope (SEM; S-4800, Hitachi High Technologies, Japan) at an acceleration voltage of 5.0 kV, and X-ray photoelectron spectroscopy (XPS) experiments were performed on a scanning X-ray microprobe (ESCALAB 250XI, Thermo Fisher Scientific, USA). The Raman spectrum data were collected using a confocal Raman (WITec, Alpha 300R, 532 nm) system with an acceleration voltage of 1.0 eV. For the post-discharge analysis of the electrodes, the disassembled cathodes from the cells were then packed in an Ar-filled glove box before they were transferred to the SEM chamber or Raman spectrometer.

### Electrochemical measurements

Galvanostatic cycling at various current densities (0.05–20.0 A g^−1^) and power analysis were used to evaluate battery performance (capacity, voltage, and power density) by using a computer-controlled battery measurement system (WBCS 3000, WonATech, Korea and VMP3 Multichannel Workstation, BioLogic). For the experiments performed, the aprotic electrolytes were tested at room temperature, and the quinary-molten salt electrolyte was tested at various operating temperatures (100–150 °C) in a lab-built heating kit. The details of the differential electrochemical mass spectrometer (DEMS) system are described elsewhere^41^. Prior to measurement of the electrochemical performance of the quinary-molten-salt-based Li–CO_2_ battery, the cell was maintained at a constant temperature (100–150 °C) to fully impregnate the quinary salt and stabilize the gas pressure (1050 Torr). Carbon dioxide gas with an isotope of carbon (^13^CO_2_) was purchased from Cambridge Isotope Laboratories, Inc. The input power required to maintain a temperature of 150 °C was ~0.6 mW.

## Supplementary information

Supplementary information

## Data Availability

The data that support the findings of this study are available from the corresponding author upon request.
