# Supplementary material for: Synergistic effect of quinary molten salts and ruthenium catalyst for high-power-density lithium-carbon dioxide cell
Source: Nat Commun. 2020 Jan 23;11:456. doi: 10.1038/s41467-019-14121-1 (PMC6978343; doi:10.1038/s41467-019-14121-1)
Supplement: Supplementary file 1 — Supplementary information [file 41467_2019_14121_MOESM1_ESM.pdf]

Supplementary Information

Synergistic effect of quinary molten salts and Ruthenium catalyst for high-power-density Lithium-carbon dioxide cell

Kyungeun Baek *et al.*

Supplementary Methods

Calculation details

The decomposition mechanism of Li_2CO_3 compound (charge process), and the formation mechanism of Li_2CO_3 with and without Ru surface (discharge process) were by density functional theory (DFT) calculation. DFT calculations were performed with the DMol^3 program[1,2]. Generalized gradient approximation (GGA) and PBEsol were used for exchange-correlation functional[3]. The effective core potentials were used for core treatment with the basis set of DNP 4.4 level. The convergence tolerances of energy, force and displacement were set to 1×10^{-5} Ha, 0.002 Ha Å^{-1}, and 0.005 Å, respectively. To include van der Waals interaction effect, Tkatchenko–Scheffler (TS) scheme was used[4]. The Brillouin-zone was sampled by a Monkhorst–Pack and *k*-point meshes for the bulk and slab models were set to (2×3×3) and (2×1×1), respectively. The COSMO solvation model was applied and the dielectric constant of quinary molten salt (ε = 5.0) was used[5,6]. Single linear synchronous transit (LST) and quadratic synchronous transit (QST) methodologies were applied to calculate transition states in reaction pathways of Li_2CO_3 decomposition, and the convergence criteria value of the root mean square (RMS) force was set to 0.003 Ha Å^{-1} [7,8].

Model systems

The unit cell structure of Li_2CO_3, which was reported from previously experimental XRD study, was optimized by DFT calculations (Supplementary Fig. 11A)[9]. The optimized lattice parameters for the monoclinic Li_2CO_3 (i.e., a = 8.25 Å, b = 4.90 Å, and c = 5.89 Å) were well matched with those of experimental crystal. To construct the surface slab models, we considered (001)-oriented three layers of the Li_2CO_3 slab model because of the most stable surface energy (Supplementary Fig. 11B)[10,11]. In all calculations for slab model, one layer on

the top was allowed to relax, while two layers at the bottom were fixed to their position to represent the bulk phase during geometry optimization calculation. The vacuum space with a height of at least 12 Å was applied to slab model. To calculate the synergistic effect of quinary molten nitrate salts and Ru nanoparticles in discharge process, we constructed a Ru surface slab model, which consisted of (101) surface observed in the experiment. The slab models for our calculations consisted of 4 atomic layers. Among four layers, two layers on the top were relaxed while two layers on the bottom were fixed to represent the bulk phase. The vacuum space was applied at least 20 Å for all slab models. For the charge balance of system, K^+, which has the largest molar ratio in molten salt, was added explicitly to vacuum space of each slab model (Supplementary Fig. 21). Note that the vacuum space was treated to be implicitly the molten salt environment by the COSMO method.

Free energy calculations

The Li^+ extraction free energy, reaction free energy, activation free energy for decomposition reactions were calculated as follows,

$$\Delta G_X = \Delta E_X + \Delta ZPE - T\Delta S \text{ (X = TRV, R, a)} \tag{1}$$

where ΔE_X represents the Li^+ extraction energy (ΔE_{TRV}) in electrochemical reaction, and the heat of reaction (ΔE_R), and activation energy (ΔE_a) for each reaction, ΔZPE is the change of zero-point vibrational enthalpy, and $-T\Delta S$ is the entropic contribution at 100 °C and 150 °C, where T is the temperature of system and ΔS is the change of entropy. Notably, the electrochemical reaction energy, which is calculated directly from the differences between total energies of before and after the extraction of lithium, is represented as the theoretical

lithiation–delithiation reaction voltage (ΔE_{TRV})[12]. This energy was calculated as follows,

$$\Delta E_{TRV} = E_{Li_xCO_3} + (2-x)E_{Li} - E_{Li_2CO_3} \ (x = 0, 1, 2) \quad (2)$$

where $E_{Li_xCO_3}$ and $E_{Li_2CO_3}$ are the total energies of a formula unit for Li_xCO_3 and Li_2CO_3 structure, respectively, and E_{Li} is the energy of one atom in the lithium metal.

For the discharge process after charge process in Supplementary Fig. 7, Gibbs free energies were calculated based on equation (1) substituting ΔZPE with the vibrational enthalpy in each temperature.

Supplementary Table 1. Summary of electrochemical performance for recently reported Li-CO_2 batteries.

Cathode materials	Electrolyte	Current density	Cycle	Ref
CNT	1 M LiTFSI in TEGDME	50 mA g^{-1}	29	13
CNT	LiTFSI in TEGDME, PVDF-HFP in NMP, HMPP-TMPET (4:5:3 wt%) (GPE)	500 mA g^{-1}	60	14
Ketjen black	1 M LiTFSI in TEGDME + LiBr	100 mA g^{-1}	38	15
N-doped graphene (Ni-NG)	1 M LiTFSI in TEGDME	100 mA g^{-1}	100	16
N-doped graphene (Cu-NG)	1 M LiTFSI in TEGDME	200 mA g^{-1}	50	17
B,N-Codoped holey graphene (BN-hG)	1 M LiTFSI in TEGDME	100 mA g^{-1}	200	18
MOF + CNT $Mn(HCOO)_2$	1 M LiTFSI in TEGDME	200 mA g^{-1}	50	19
NiO + CNT	1 M LiTFSI in TEGDME	50 mA g^{-1}	42	20
RuO_2 + CNT	0.25 M $LiCF_3SO_3$ in TEGDME	50 mA g^{-1}	30	21
Fiber-shaped N-CNTs @ Ti	1 M LiTFSI in TEGDME	250 mA g^{-1}	45	22
Ru + Super P	$LiCF_3SO_3$ in TEGDME	100 mA g^{-1}	80	23
RuO_2/LDO + Super P	1 M LiTFSI in TEGDME	200 mA g^{-1}	60	24
Ir + CNFs	1 M LiTFSI in TEGDME	50 mA g^{-1}	45	25
IrO_2/d-MnO_2–carbon cloth	1 M $LiClO_4$ in TEGDME	400 mA g^{-1}	378	26
Graphene	1 M LiTFSI in TEGDME	50 mA g^{-1}	20	27
CNT	Poly(methacrylate)/poly(ethylene glycol)–$LiClO_4$–3wt%SiO_2) composite polymer electrolyte (CPE)	100 mA g^{-1}	100	28
CoPPc	1 M LiTFSI in TEGDME	0.05 mA cm^{-2}	50	29
Ru-Cu-graphene	1 M LiTFSI in TEGDME	400 mA g^{-1}	100	30
Mo_2C + CNT	1 M $LiCF_3SO_3$ in TEGDME	0.02 mA	40	31
Ru + CNT	1 M LiTFSI in TEGDME	200 mA g^{-1}	200	32
P-Mn_2O_3 + Ketjen black	0.5 M $LiClO_4$ in DMSO	50 mA g^{-1}	45	33
N-doped carbon nanofibers (IrNSs-CNFs)	1 M LiTFSI in TEGDME	500 mA g^{-1}	400	34
Anatase TiO_2 nanoparticle/ CNT–CNF composite	1 M LiTFSI in DMSO	0.05 mA cm^{-2}	25	35
RuP_2-NPCFs	1 M LiTFSI in TEGDME	200 mA g^{-1}	200	36
NiFe @ NC/PPC	1 M $LiCF_3SO_3$ in TEGDME	0.05 mA cm^{-2}	109	37
Ru @ CNFs	1 M LiTFSI in TEGDME	100 mA g^{-1}	50	38

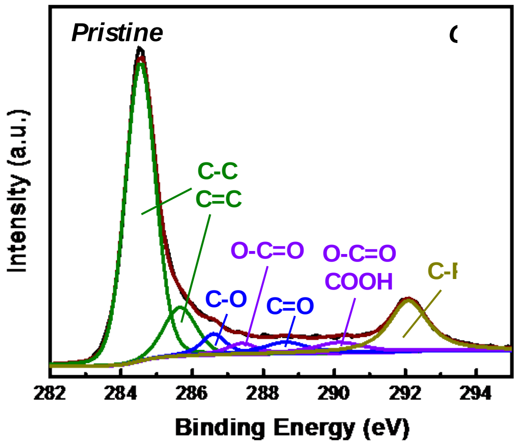

Supplementary Figure 1 | Characterization of pristine Ketjen black cathode. High-resolution XPS C1s spectra of pristine Ketjen black carbon cathode.

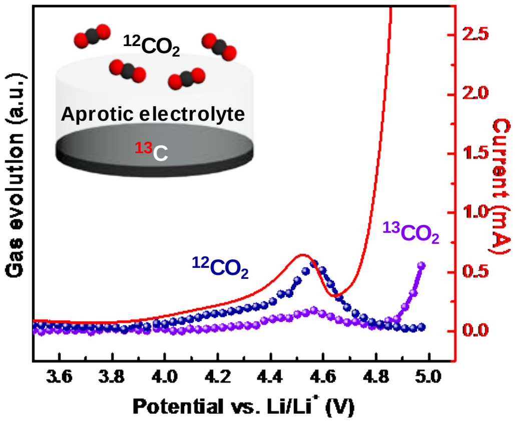

Supplementary Figure 2 | Linear-sweep voltammetry profile (at a scan rate of 0.1 mV s^{-1}) and complementary gas analysis of 1 M LiTFSI in TEGDME containing a cell with a ^{13}C cathode for a 1 mAh cell discharged in a CO_2 environment.

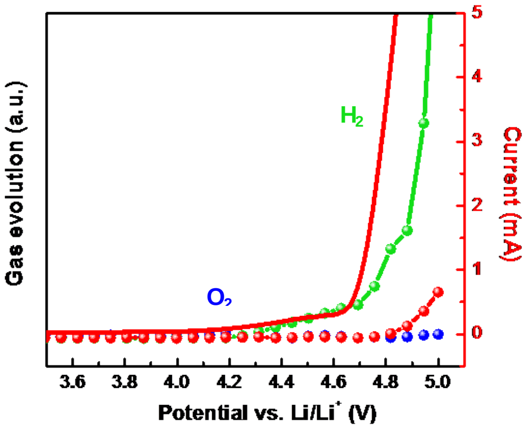

Supplementary Figure 3| Aprotic solvent decomposition potential analysis using linear sweep voltammetry with *in situ* DEMS. Linear sweep voltammetry with *in situ* DEMS results of a fresh cell containing 1 M LiTFSI in TEGDME with scan rate of 0.1 mV s^{-1}.

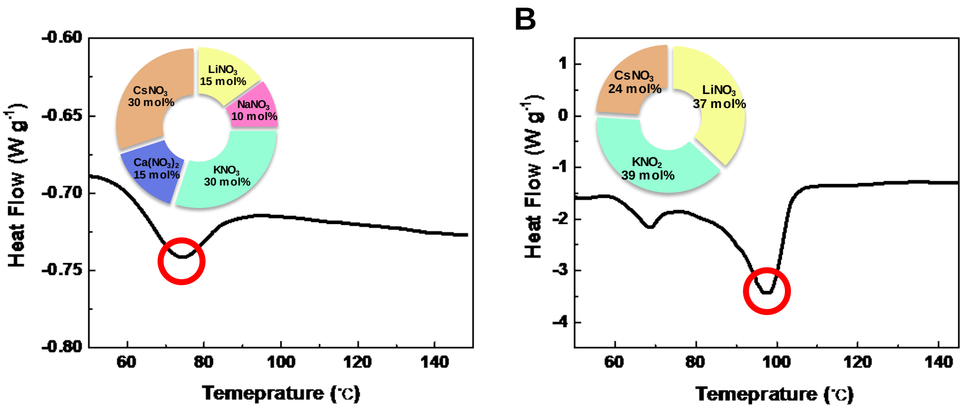

Supplementary Figure 4| Quinary and ternary molten salt electrolyte. **A-B** Differential scanning calorimeter (DSC) profile of the quinary eutectic molten salt (**A**) and ternary eutectic molten salt (**B**) obtained at a scanning rate of 5.0 °C min^{-1} with schematic illustration of eutectic molten salt electrolyte containing salt portions (inset images).

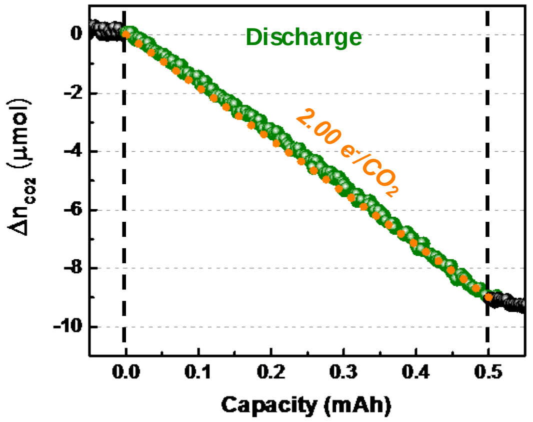

Supplementary Figure 5 | CO_2 gas consumption measurement in the nitrate-based electrolyte. *In situ* DEMS result of CO_2 gas consumption during discharge using quinary molten salt electrolyte with Super P carbon cathode at 100 °C (200 µA for 2 h 30 min). The orange dots indicate the ideal electrons-to-CO_2 ratio of 2.0.

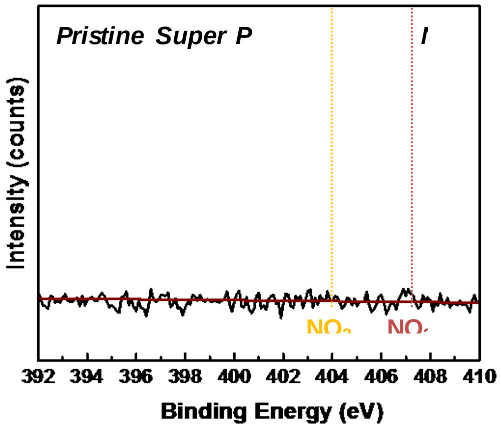

Supplementary Figure 6 | High-resolution XPS N1s spectra of the Super P carbon cathode before discharging.

Supplementary Table 2. Thermodynamic energy terms in the Gibbs free energy of the reaction.

Electrochemical reaction	Temp. (°C)	ΔH (eV)	$-T\Delta S$ (eV)	ΔG (eV)	Discharge potential versus Li/Li^+ (V)
$2Li^+ + CO_2 + 2e^- + NO_3^- \rightarrow Li_2CO_3 + NO_2^-$	100	-5.23	0.40	-4.83	2.42
	150	-5.21	0.43	-4.78	2.39

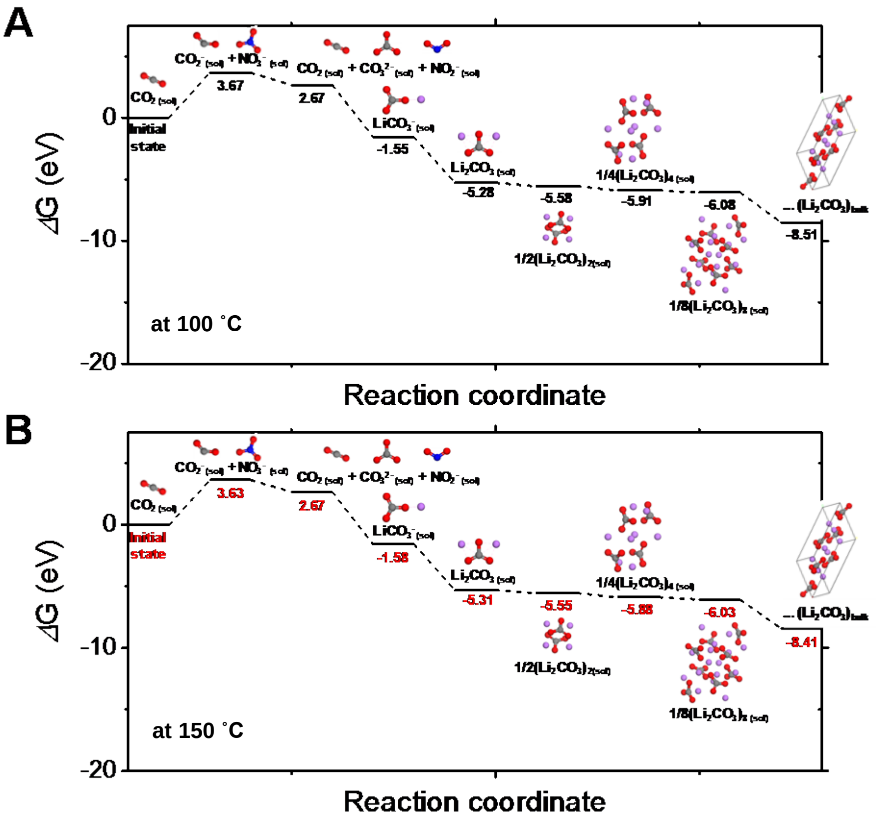

Supplementary Figure 7 | Free energy diagrams of the discharge process at 100 and 150 °C. **A–B** The electrochemical reaction starts from CO_2. The black numbers in **A** and the red numbers in **B** below each energy state represent the relative free energies compared to each initial state at 100 and 150 °C. Oxygen, carbon, nitrogen, and lithium atoms are colored in red, gray, blue, and purple, respectively.

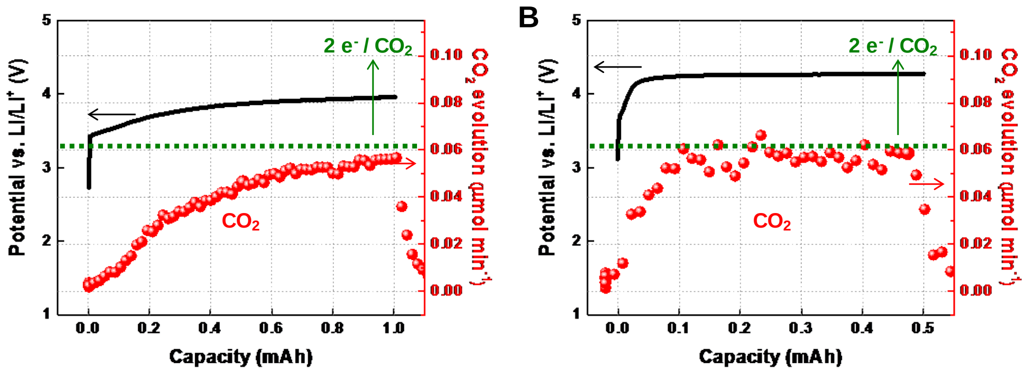

Supplementary Figure 8 | A Galvanostatic charge plot and the corresponding DEMS results of the deep discharged Li-CO_2 cell with quinary molten salt electrolyte at 100 °C. **B** Galvanostatic and corresponding DEMS measurements after reaching a CO_2 evolution rate of 0.06 μmol min^{-1} in **A** and intentionally stopping and restarting the Li–CO_2 cell (applied current: 0.2 mA).

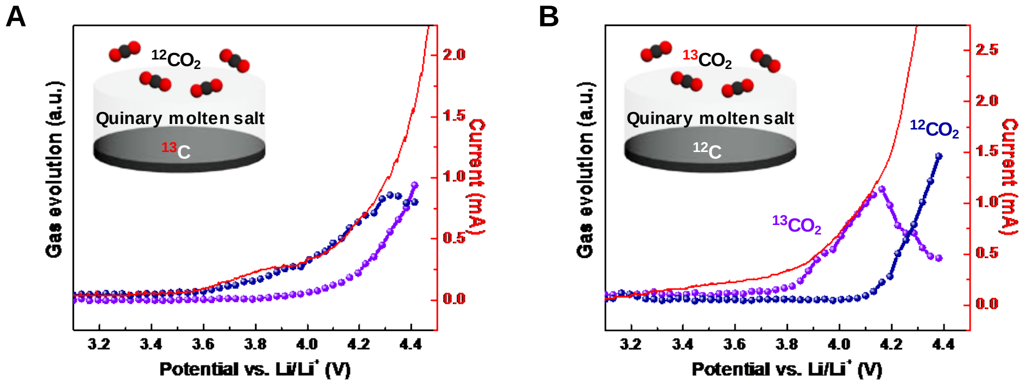

Supplementary Figure 9 | A–B Linear-sweep voltammetry profiles (at a scan rate of 0.1 mV s^{-1}) and complementary gas analysis of a quinary molten salt electrolyte containing a cell with $^{12}CO_2$–^{13}C (**A**) and $^{13}CO_2$–^{12}C (**B**) cathodes for a cell discharged in a CO_2 environment at 100 °C.

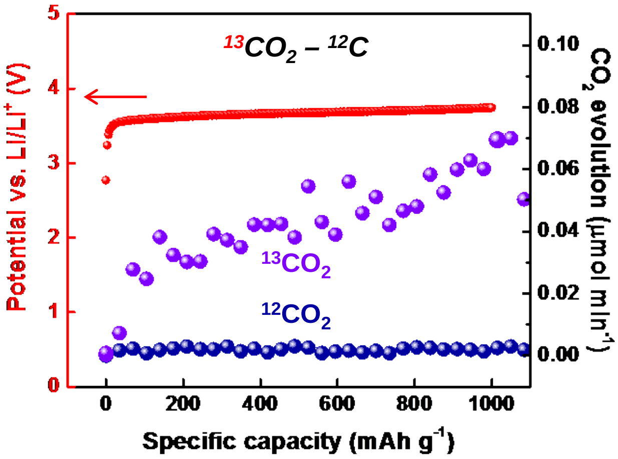

Supplementary Figure 10 | Galvanostatic charge plot (applied current density: 0.4 A g^{-1}) and corresponding DEMS results of a Li–CO_2 cell containing a quinary molten salt electrolyte with a $^{13}CO_2$–^{12}C cathode discharged in a CO_2 environment at 100 °C.

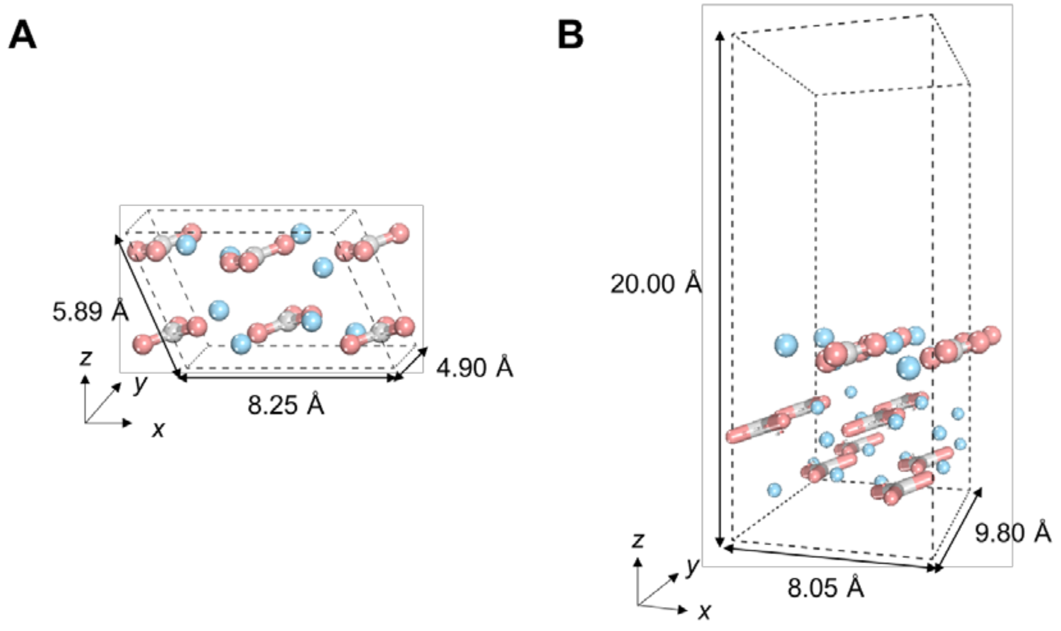

Supplementary Figure 11 | Li_2CO_3 model systems employed for reaction step calculation. **A-B** Unit cell structure (**A**) for monoclinic Li_2CO_3 (space group - *C*2/*c*), and slab model (**B**) of three layered Li_2CO_3 on (001) direction. Carbon, oxygen, and lithium atoms are colored in light gray, pink, and sky blue, respectively. For clear view in **B**, top layer is presented by ball-and-stick style, and bottom two layers, which are fixed in position, are displayed in stick style.

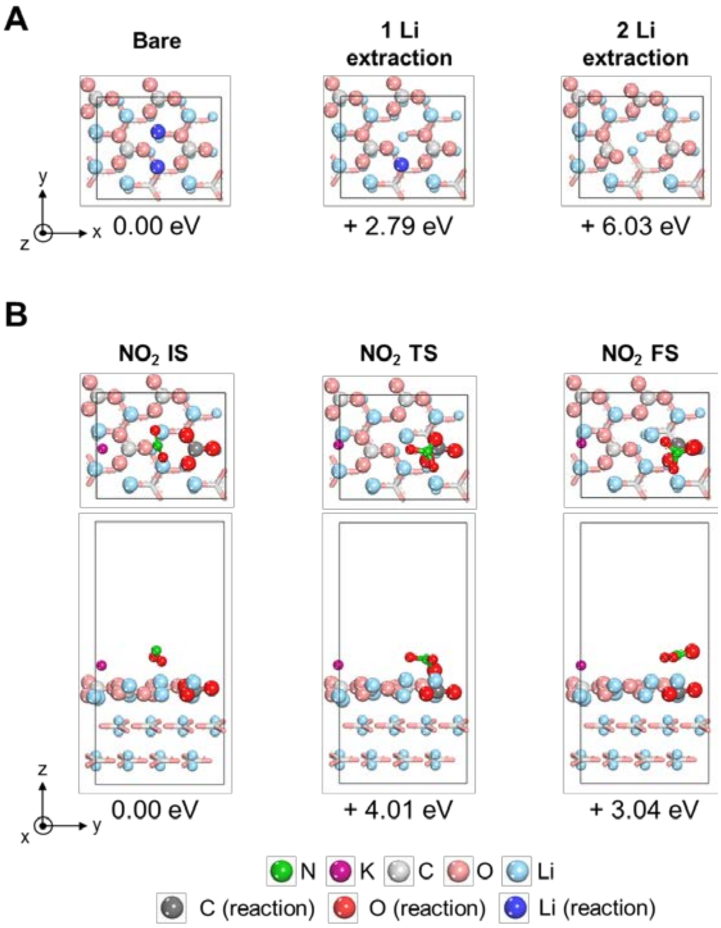

Supplementary Figure 12 | Comparison of the electrochemical reaction step and reaction step of Li_2CO_3 decomposition on the surface at 100 °C. **A** Optimized configurations of the extraction of Li reactions. **B** Optimized configurations of the reaction mechanism between CO_3^{2-} and NO_2^- to produce CO_2 and NO_3^-. The states and relative energies are written in the top and bottom of each figure. NO_2 IS, NO_2 TS, and NO_2 FS represent initial state, transition state, and final state, respectively. Nitrogen, potassium, carbon, oxygen, and lithium atoms are colored in green, purple, light gray, pink, and sky blue. And, for the clear view, the carbon, oxygen, and lithium atoms which participate in the reaction are colored in dark gray, red, and blue.

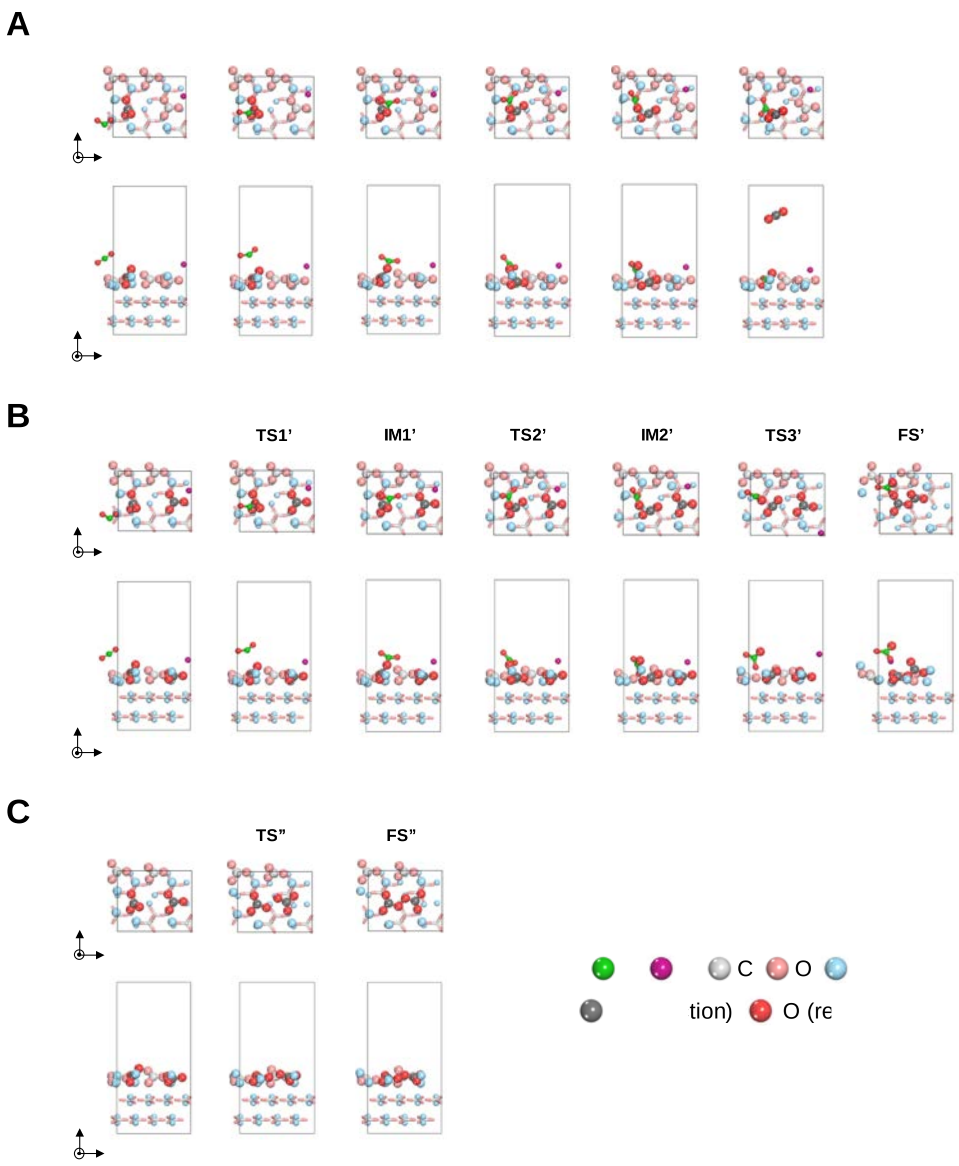

Supplementary Figure 13 | Optimized configurations of three plausible pathways for reaction step of Li_2CO_3 decomposition. **A**-**C** Reaction path a (**A**), reaction path b (**B**), and reaction path c (**C**) which produce NO_3^- and CO_2, NO_3^- and $C_2O_5^{2-}$, and $C_2O_6^{2-}$, respectively. The names of states are written on the top of each figure. IS, IM, TS, and FS in each reaction mechanism represent the initial state, intermediate state, transition state, and final state, respectively. Color scheme is same with Supplementary Fig. 12.

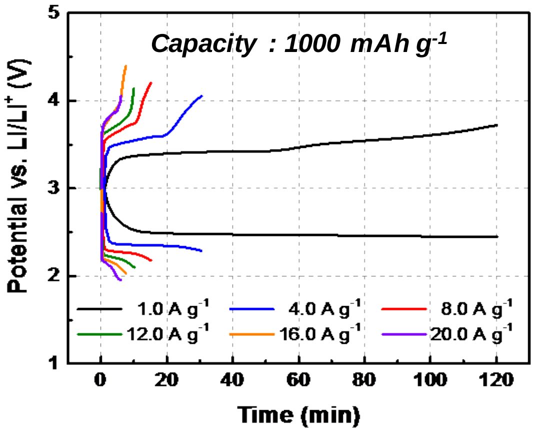

Supplementary Figure 14 | Plot of the potential versus time of a Li–CO_2 cell with a quinary molten salt electrolyte at current densities ranging from 1.0 to 20.0 A g^{-1} at 150 °C.

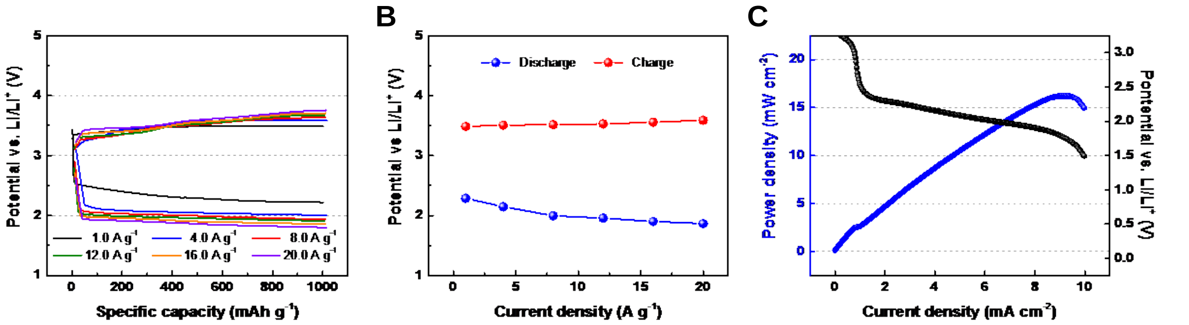

Supplementary Figure 15 | Electrochemical characterization of a Li–CO_2 cell with a ternary molten salt electrolyte. **A–B** Galvanostatic discharge–charge profile (**A**) and plot of the discharge–charge overpotential (**B**) measured at 500 mAh g^{-1} for a Li–CO_2 cell with a ternary molten salt electrolyte at current densities ranging from 1.0 to 20.0 A g^{-1} at 150 °C. **C** Polarization and power density curves of a Li–CO_2 cell using a ternary molten salt electrolyte at a scan rate of 0.01 mA s^{-1} and 150 °C.

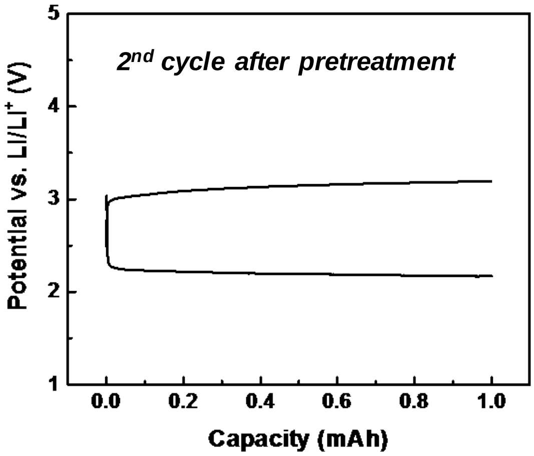

Supplementary Figure 16 | Galvanostatic discharge–charge plot of a Li–CO_2 cell using a quinary molten salt electrolyte for the 2nd cycle after a pretreatment at 150 °C (applied current: 0.2 mA).

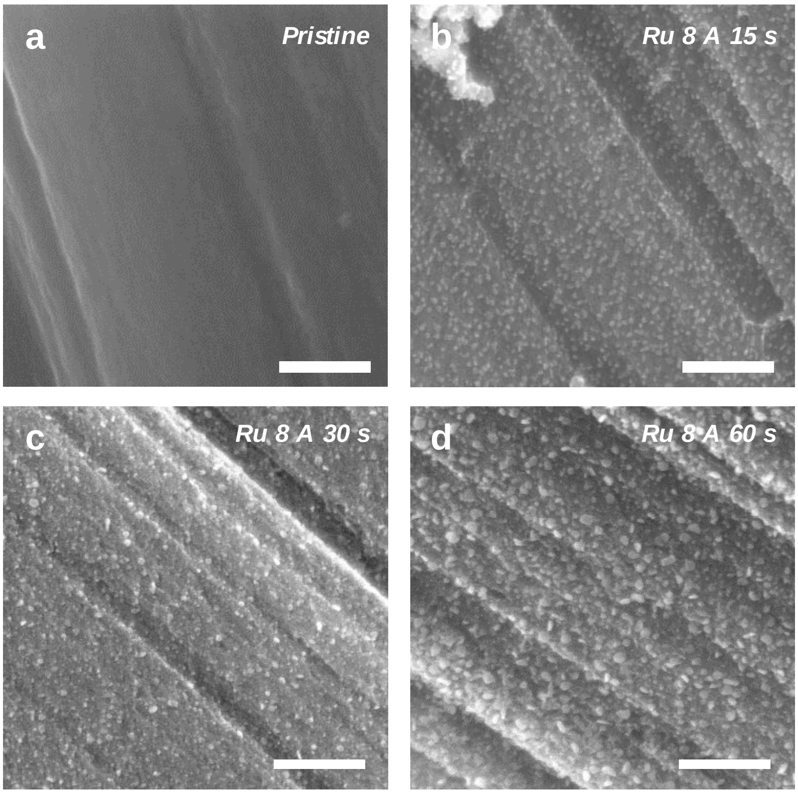

Supplementary Figure 17 | SEM images of Ru nanoparticles on carbon cathodes. **A**-**D** SEM images for pristine carbon cathode (**A**) and Ru doped carbon cathode using joule heating method applied current at 8 A for 15 s (**B**), 30 s (**C**), and 60 s (**D**).

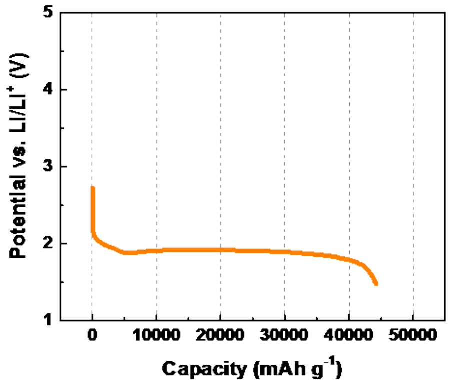

Supplementary Figure 18 | The discharge profile of Li-CO_2 cell with quinary molten salt electrolyte and Ru/C cathode. Galvanostatic profile of the Li-CO_2 battery with quinary molten salt electrolyte and Ru/C cathode at 10.0 A g^{-1} within a voltage cut-off 1.5 V.

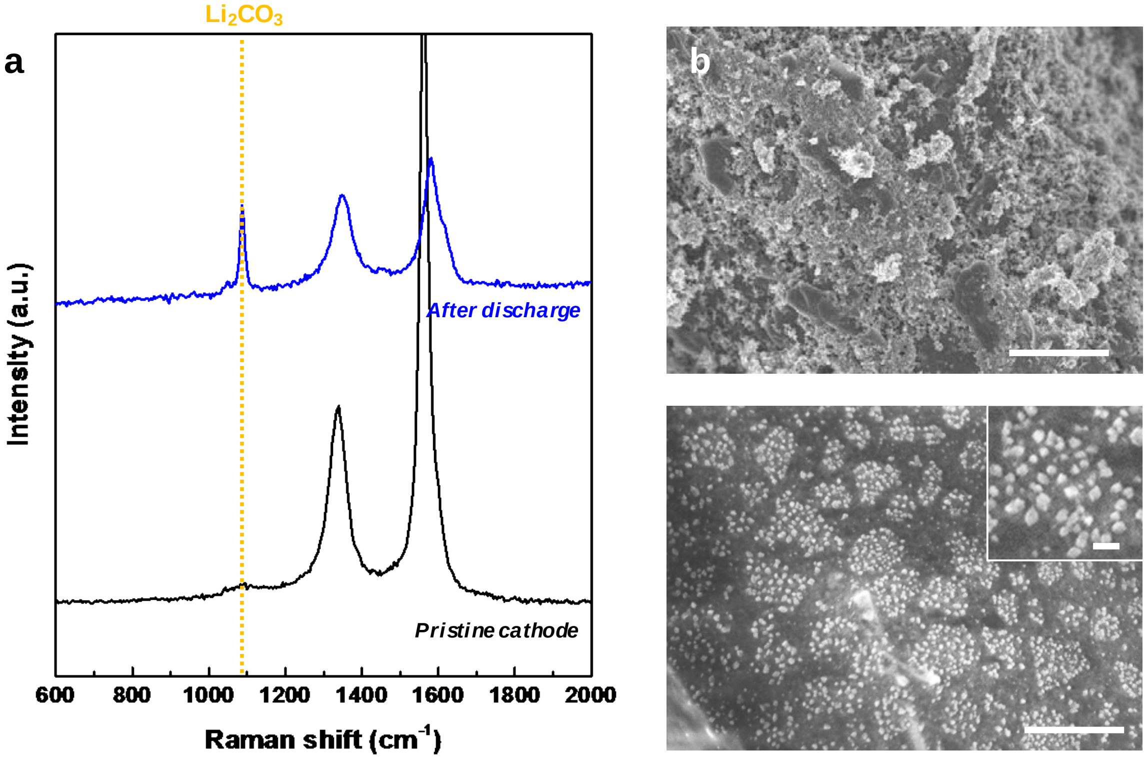

Supplementary Figure 19 | Discharge product for a Li–CO_2 cell with an Ru/C cathode and quinary molten salt electrolyte at 150 °C. **A** Raman spectra of the Ru/C cathode before (black line) and after (blue line) discharging a 1 mAh cell in a CO_2 environment. **B–C** Top-view SEM images of the Ru/C cathode before (**B**) and after (**C**) discharging a 1 mAh cell in a CO_2 environment. The cathode was rinsed with an *N*-methylacetamide solvent in an Ar-filled glove box.

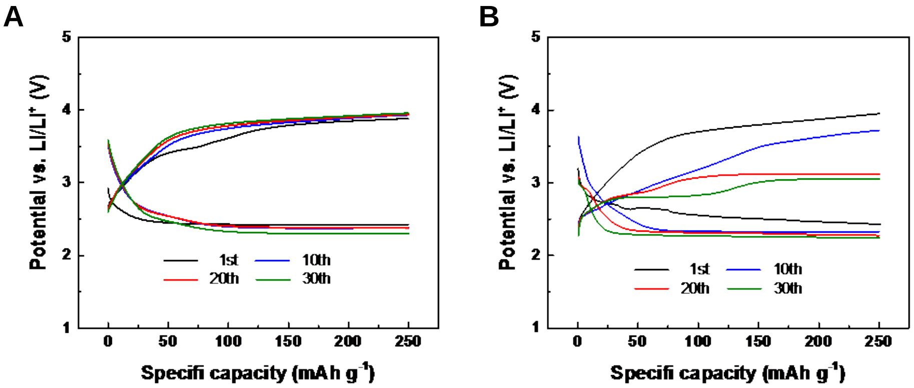

Supplementary Figure 20| Cycling performance for a Li–CO_2 cell with an aprotic electrolyte. **A**–**B** Galvanostatic discharge–charge profiles of a Li–CO_2 battery containing a 1 M LiTFSI/TEGDME electrolyte with pristine carbon (**A**) and Ru on carbon (**B**) cathodes at a current density of 50.0 mA g^{-1}.

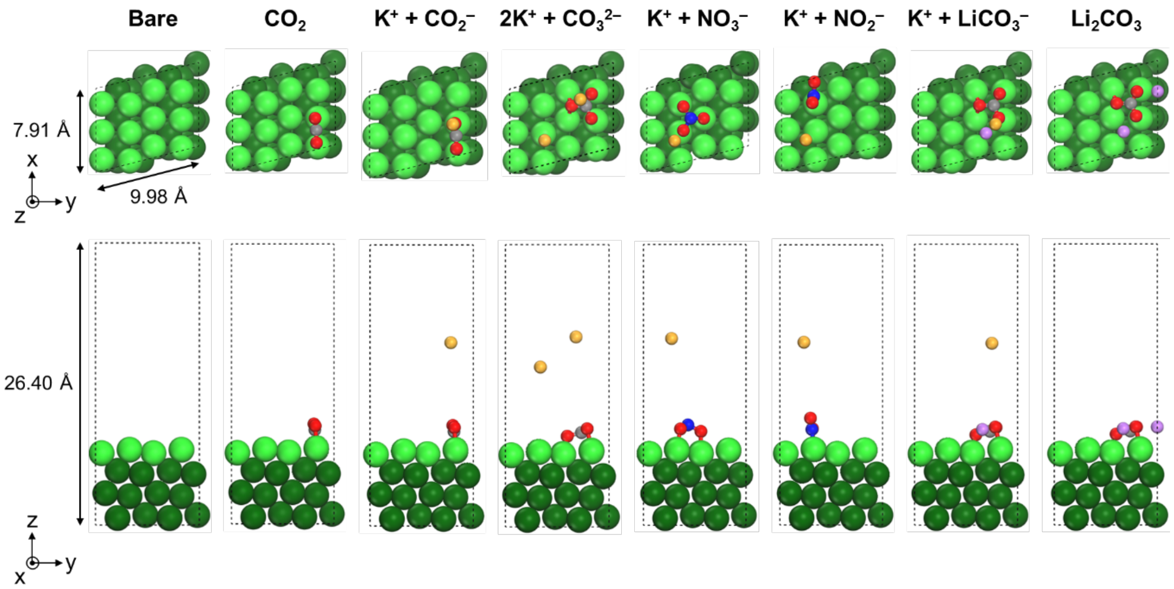

Supplementary Figure 21 | Optimized configurations of each molecule adsorbed on Ru (101) surface for discharge process. The empty region was treated by the COSMO method to impose the explicit molten salt phase. To balance an atomic charge, K^+ ion was added in explicit solvent phase of each model wherever necessary. Nitrogen, carbon, oxygen, ruthenium, lithium, and potassium atoms are colored in blue, light gray, red, dark cyan, purple, and yellow, respectively. For the clear view, Ru atoms in the top layer were colored in mint green.

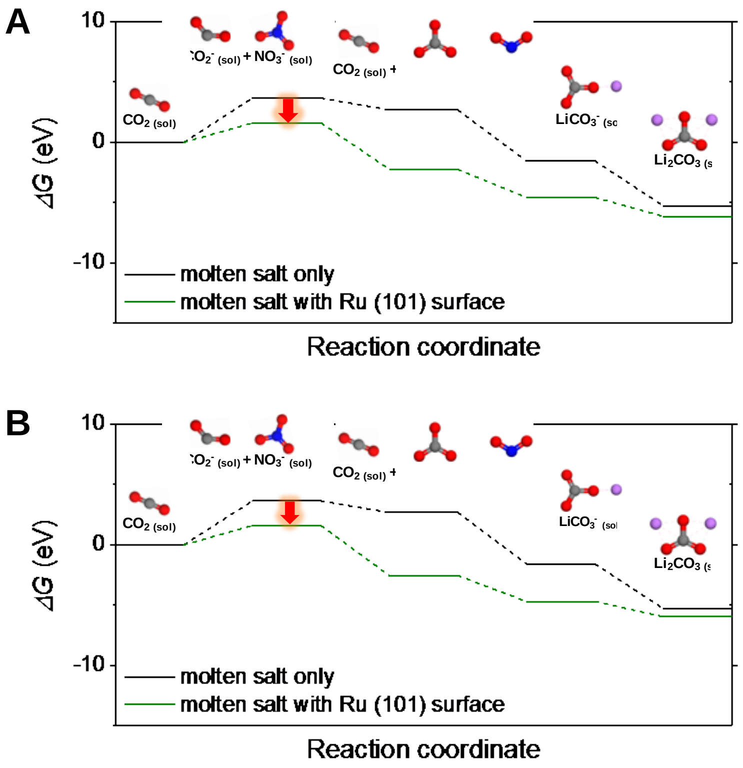

Supplementary Figure 22 | Free energy diagrams of discharge process from CO_2 reduction to one Li_2CO_3 formation at 100 °C and 150 °C. Electrochemical reaction starts from CO_2 at 100 °C (**A)** and 150 °C (**B)**, respectively. The black and green numbers in each **A** and **B** represent the relative free energies compared to each initial state of molten salt only and Ru (101) surface. Oxygen, carbon, nitrogen, lithium atoms are colored in red, gray, blue, and purple, respectively. Red arrow and number represent the change of ΔG in potential determining step.

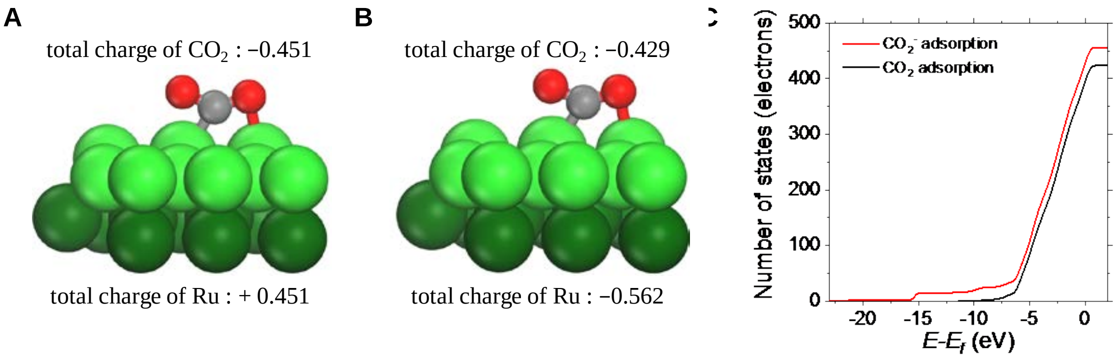

Supplementary Figure 23 | Atomic charges and configurations of CO_2–Ru surface (**A**) and CO_2^{-}–Ru surface (**B**). Integrated DOS of all Ru atoms in CO_2–Ru surface and CO_2^{-}–Ru surface (**C**).

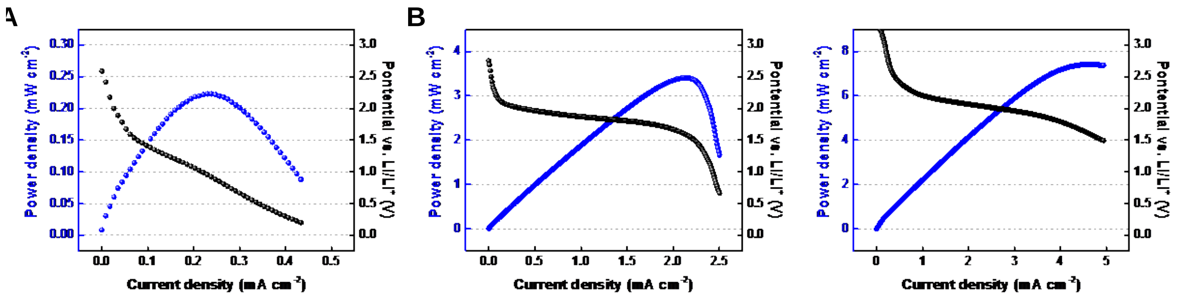

Supplementary Figure 24 | Power density profiles of Li-CO_2 cell with quinary molten salt. **A-C** Polarization and power density curves of quinary molten salt at 100 °C (**A**), 110 °C (**B**), and 130 °C (**C**) with a carbon cathode at a scan rate of 0.01 mA s^{-1}.

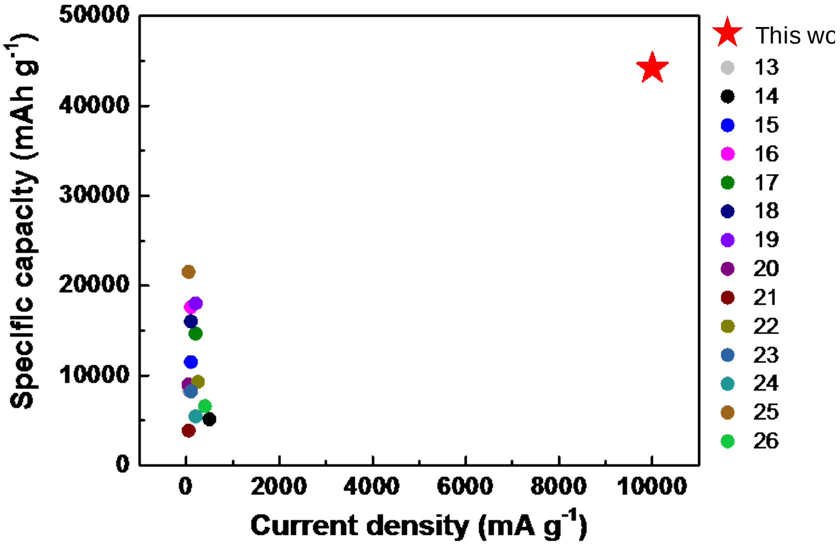

Supplementary Figure 25 | Summary of the current density and specific capacity for recent progress Li-CO_2 batteries.

Supplementary References

1. Delley, B. An all -electron num
for polyatomic molecules. *J. Chem. Phys.* **92**, 508–517 (1990).
2. Delley, B. From molecules to solids with the Dmol^3 approach. *J. Chem. Phys.* **113**, 7756–7764 (2000).
3. Perdew, J. P., Ruzsinszky, A., Csonka, G. I., Vydrov, O. A., Scuseria, G. E., Constantin, L. A., Zhou, X. & Burke, K. Restoring the density-gradient expansion for exchange in solids and surfaces. *Phys. Rev. Lett.* **100**, 136406 (2008).
4. Tkatchenko, A. & Xcheffler, M. Accurate molecular van der Waals interactions from ground-state electron density and free-atom reference data. *Phys. Rev. Lett.* **102**, 073005 (2009).
5. Klamt, A. & Schüürmann, G. COSMO: a new approach to dielectric screening in solvents with explicit expressions for the screening energy and its gradient. *J. Chem. Soc. Perkin Trans. 2* **5**, 799–805 (1993).
6. Looyenga, H. Dielectric constants of homogeneous mixture. *Mol. Phys.* **9**, 501–511 (1965).
7. Bell, S. & Crighton, J. S. Locating transition states. *J. Chem. Phys.* **80**, 2464–2475 (1984).
8. Halgren, T. A. & Lipscomb, W. N. The synchronous-transit method for determining reaction pathways and locating molecular transition states. *Chem. Phys. Lett.* **49**, 225–232 (1977).
9. Dffenberger, H. & Zemann, J. ζristallographie. Z. Kristallogr, *Cryst. Mater.* **150**, 133–138 (1979).
10. Liu, Z., Qi, Y., Lin, Y. X., Chen, L., Lu, P. & Chen, L. Q. Interfacial study on solid electrolyte interphase at Li metal anode: implication for Li dendrite growth. *J. Electrochem. Soc.* **163**, A592–A598 (2016).
11. Ling, C., Zhang, R., Takechi, K. & Mizuno, F. Intrinsic Barrier to Electrochemically Decompose Li_2CO_3 and LiOH. *J. Phys. Chem. C* **118**, 26591−26598 (2014).
12. Kalantarian, M. M., Asgari, S. & Mustarelli, P. Theoretical investigation of Li_2MnSiO_4 as a cathode material for Li-ion batteries: a DFT study. *J. Mater. Chem. A* **1**, 2847–2855 (2013).
13. Zhang, X., Zhang, Q., Zhang, Z., Chen, Y., Xie, Z., Wei, J. & Zhou, Z. Rechargeable Li–CO_2 batteries with carbon nanotubes as air cathode. *Chem. Commun.* **51**, 14636–14639 (2015).
14. Li, C., Guo, Z., Yang, B., Liu, Y., Wang, Y. & Xia, Y. A rechargeable Li CO_2 battery with a gel polymer electrolyte. *Angew. Chem. Int. Ed.* **56**, 9126–9130 (2017).
15. Wang, X. G., Wang, C., Xie, Z., Zhang, X., Chen, Y., Wu, D. & Zhou, Z. Improving electrochemical performances of rechargeable Li−CO_2 batteries with an electrolyte redox mediator. *Chem. Electrochem.* **4**, 2145–2149 (2017).
16. Zhang, Z., Wang, X. G., Zhang, X., Xie, Z., Chen, Y. N., Ma, L., Peng, Z. & Zhou, Z. Verifying the rechargeability of Li CO_2 batteries on working cathodes of Ni nanoparticles highly dispersed on N doped graphene. *Adv. Sci.* **5**, 1700567 (2018).
17. Zhang, Z., Zhang, Z., Liu, P., Xie, Y., Cao, K. & Zhou, Z. Identification of cathode stability in Li–CO_2 batteries with Cu nanoparticles highly dispersed on N-doped graphene. *J. Mater. Chem. A* **6**, 3218–3223 (2018).

18. Qie, L., Lin, Y., Connell, J. W., Xu, J. & Dai, L. Highly rechargeable lithium–CO_2 batteries with a boron- and nitrogen-codoped holey-graphene cathode. *Angew. Chem. Int. Ed.* **56**, 6970–6974 (2017).
19. Li, S., Dong, Y., Zhou, J., Liu, Y., Wang, J., Gao, X., Han, Y., Qi, P. & Wang, B. Carbon dioxide in the cage: manganese metal–organic frameworks for high performance CO_2 electrodes in Li–CO_2 batteries. *Energy Environ. Sci.* **11**, 1318–1325 (2018).
20. Zhang, X., Wang, C., Li, H., Wang, X.-G., Chen, Y.-N., Xie, Z. & Zhou, Z. High performance Li–CO_2 batteries with NiO–CNT cathodes. *J. Mater. Chem. A* **6**, 2792–2796 (2018).
21. Bie, S., Du, M., He, W., Zhang, H., Yu, Z., Liu, J., Liu M., Yan. W., Zhou, L. & Zou, Z. Carbon Nanotube@RuO_2 as a High Performance Catalyst for Li–CO_2 Batteries. *ACS Appl. Mater. Interfaces* **11**, 5146–5151 (2019).
22. Li, Y., Zhou, J., Zhang, T., Wang, T., Li, X., Jia, Y., Cheng, J., Guan, Q., Liu, E. & Peng, H. Highly surface-wrinkled and N-doped CNTs anchored on metal wire: a novel fiber-shaped cathode toward high-performance flexible Li–CO_2 batteries. *Adv. Funct. Mater.* **29**, 1808117 (2019).
23. Yang, S., Qiao, Y., He, P., Liu, Y., Cheng, Z., Zhu, J.–j. & Zhou, H. A reversible lithium–CO_2 battery with Ru nanoparticles as a cathode catalyst. *Energy Environ. Sci.* **10**, 972–978 (2017).
24. Xu, S.–M., Ren, Z.–C., Liu, X., Liang, X., Wang, K.–X. & Chen, J.–S. Carbonate decomposition: low-overpotential Li-CO_2 battery based on interlayer-confined monodisperse catalyst. *Energy Stor. Mater.* **15**, 291–298 (2018).
25. Wang, C., Zhang, Q., Zhang, X., Wang, X. G., Xie, Z. & Zhou, Z. Fabricating Ir/C nanofiber networks as free-standing air cathodes for rechargeable Li–CO_2 batteries. *Small* **14**, 1800641 (2018).
26. Mao, Y., Tang, C., Tang, Z., Xie, J., Chen, Z., Tu, J., Cao, G. & Zhao, X. Long-life Li–CO_2 cells with ultrafine IrO_2-decorated few-layered δ-MnO_2 enabling amorphous Li_2CO_3 growth. *Energy Stor. Mater.* **18**, 405–413 (2018).
27. Zhang, Z., Zhang, Q., Chen, Y., Bao, J., Zhou, X., Xie, Z., Wei, J. & Zhou, Z. The First Introduction of Graphene to Rechargeable Li–CO_2 Batteries. *Angew. Chem. Int. Ed.* **54**, 6550–6553 (2015).
28. Hu, X., Li, Z. & Chen, J. Flexible Li–CO_2 batteries with liquid-free electrolyte. *Angew. Chem. Int. Ed.* **129**, 5879–5883 (2017).
29. Chen, J., Zou, K., Ding, P., Deng, J., Zha, C., Hu, Y., Zhao, X., Wu, J., Fan, J. & Li, Y. Conjugated cobalt polyphthalocyanine as the elastic and reprocessable catalyst for flexible Li–CO_2 batteries. *Adv. Mater.* **31**, 1805484 (2018).
30. Zhang, Z., Yang, C., Wu, S., Wnag, A., Zhao, L., Zhai, D., Ren, B., Cao, K. & Zhou, Z. Exploiting Synergistic Effect by Integrating Ruthenium–Copper Nanoparticles Highly Co [illegible] Dispersed on Graphene as Ef [illegible] –CO_2 Batteries. *Adv. Energy. Mater.* **9**, 1802805 (2019).
31. Hou, Y., Wang, J., Liu, L., Liu, Y., Chou, S., Shi, D., Liu, H., Wu, Y., Zhang, W. & Chen, J. Mo_2C/CNT: An Efficient Catalyst for Rechargeable Li–CO_2 Batteries. *Adv. Funct. Mater.* **27**, 1700564 (2017).
32. Xu, S., Chen, C., Kuang, Y., Song, J., Gan, W., Liu, B., Hitz, E. M., Connell, J. W., Lin, Y. & Hu, L.. Flexible lithium–CO_2 battery with ultrahigh capacity and stable cycling. *Energy Environ. Sci.* **11**, 3231–3237 (2018).

33. Ma, W., Lu, S., Lei, X., Liu, X. & Ding, Y.. Porous Mn_2O_3 cathode for highly durable Li–CO_2 batteries. *J. Mater. Chem. A* **6**, 20829–20835 (2018).
34. Xing, Y., Yang, Y., Li, D., Luo, M., Chen, N., Ye, Y., Quan, J., Li, L., Yang, D. & Wu, F. Crumpled Ir nanosheets fully covered on porous carbon nanofibers for long‐life rechargeable Lithium–CO_2 batteries. *Adv. Mater.* **30**, 1803124 (2018).
35. Ripes, R., Bhargav, A. & Manthiram, A. Nanostructured anatase titania as a cathode catalyst for Li–CO_2 batteries. *ACS Appl. Mater. Interfaces* **10**, 37119–37124 (2018).
36. Guo, Z., Li, J., Qi, H., Sun, X., Li, H., Tamirat, A. G., Liu, J., Wang, Y. & Wang, L. A highly reversible long‐life Li–CO_2 battery with a RuP_2‐based catalytic cathode. *Small* 1803246 (2018).
37. Liang, H., Zhang, Y., Chen, F., Jing, S., Yin, S. & Tsiakaras, P. A novel NiFe@NC-functionalized N-doped carbon microtubule network derived from biomass as a highly efficient 3D free-standing cathode for Li-CO_2 batteries. *Appl. Catal., B* **244**, 559–567 (2019).
38. Qiao, Y., Xu, S., Liu, Y., Dai, J., Xie, H., Yao, Y., Mu, X., Chen, C., Kline, D., Hitz, E., Liu, B., Song, J., He, P., Zachariah, M. & Hu, L. Transient, in situ synthesis of ultrafine ruthenium nanoparticles for a high-rate Li–CO_2 battery. *Energy Environ. Sci.* **12**, 1100–1107 (2019).
